# Determination of central pain modulation biomarkers to characterise patients with cancer and refractory pain: protocol for a cross-sectional study

**DOI:** 10.1136/bmjopen-2026-121471

**Published:** 2026-10-07

**Authors:** Lara Rubal-Otero, Natalia Martinez-Rodriguez, Margarita Álvarez-Rodríguez, Alberto González-Villar, María T Carrillo-de-la-Peña, Painless Consortium, D. Yarnitzsky

**Affiliations:** 1 FIDIS, Santiago de Compostela, Spain; 2 IIS Galicia Sur, Vigo, Spain; 3 Universidad de Santiago de Compostela, Santiago de Compostela, Spain; 4 Clinical Psychology and Psychobiology, University of Santiago de Compostela, Santiago de Compostela, Spain

**Keywords:** Cancer Pain, ONCOLOGY, Electroencephalography

## Abstract

**Introduction:**

Pain is one of the most prevalent and disabling oncological symptoms, but its underlying mechanisms remain poorly understood. Most research has focused on peripheral mechanisms, while central pain modulation has been poorly explored. Quantitative sensory testing (QST) and electroencephalography (EEG) provide non-invasive approaches to assess central pain processing but the evidence using those approaches in patients with cancer is limited. This study protocol aims to identify and validate central biomarkers of pain perception and modulation (QST and EEG) and the role of moderator variables to better characterise patients with cancer pain.

**Methods and analysis:**

This study protocol describes a multicentre, cross-sectional, case–control study that will include a minimum of 470 ambulatory patients with cancer, including 313 with oncological pain and 157 without pain. Participants will be recruited taking into account age, sex and cancer type (lung, breast, pancreas, prostate and colon). All participants will perform a single face-to-face assessment that will include sociodemographic and clinical data, QST (pain modulation and inhibitory pain mechanisms, temporal summation and pain thresholds) and EEG (at rest and in response to thermal nociceptive stimuli) assessment and administration of questionnaires (pain, quality of life, fatigue, sleep, mood, etc). Statistical modelling and machine learning approaches will be used to investigate pain modulation biomarkers, their association with age and sex and to characterise patients with cancer pain.

**Ethics and dissemination:**

This study has received approval from all local ethics committees of the participating clinical sites. Results will be disseminated through high-impact peer-reviewed journals, international conferences and open data repositories, in compliance with General Data Protection Regulation (GDPR) and FAIR (Findable, Accessible, Interoperable, and Reusable) principles, and following the STROBE (Strengthening the Reporting of Observational Studies in Epidemiology) statement for observational case–control studies.

**Trial registration number:**

ISRCTN35887998.

**STRENGTHS AND LIMITATIONS OF THIS STUDY**

- This study makes a comprehensive assessment, using objective biomarkers to characterise pain processing and modulation in cancer.
- The large sample and multicentre design increases external validity and supports the development of a robust normative database of pain modulation measures across different healthcare settings.
- The protocol applies harmonised and standardised procedures for quantitative sensory testing, electroencephalography acquisition and questionnaire administration, enhancing reproducibility and comparability across sites.
- The inclusion of dynamic pain modulation indices (eg, conditioned pain modulation, temporal summation of pain and offset analgesia) allows mechanistic profiling beyond static sensory thresholds.
- The cross-sectional design limits causal inference and the assessment of longitudinal changes in pain mechanisms or treatment effects over time.

## Introduction

### Background

 Pain is highly prevalent and is one of the most debilitating symptoms, with a negative impact on the well-being of patients with cancer. It significantly impairs their quality of life, daily activities and social interactions.^1 2^ Overall pain prevalence remains high throughout the cancer trajectory (44.5%), increasing to approximately 55% in patients with advanced, metastatic or terminal disease.^2^ Although the detection and survival of patients with cancer have improved in recent years, cancer pain management continues to be a challenge and, compared with other pain conditions, the mechanisms underlying cancer-related pain remain poorly understood.^3^ Following current pain taxonomy,^4^ nociceptive, neuropathic and nociplastic pain mechanisms may be involved in oncological pain. Altered central pain processing and pain modulation are considered key mechanisms underlying nociplastic pain, and thus a better understanding of these mechanisms may improve the characterisation of cancer-related pain and support the identification of objective pain biomarkers.

It is possible to assess central mechanisms involved in pain processing and pain modulation using quantitative sensory testing (QST) procedures^5^ and brain electrical activity recordings—electroencephalography (EEG).^6^ Although quantifying the function of descending pain modulatory pathways using QST would improve our understanding of cancer pain,^5^ the available evidence is limited. Most of the existing research has focused on the study of peripheral mechanisms. Some studies have used QST in patients with chemotherapy-induced peripheral neuropathy, showing abnormal responses (mostly thresholds) to a variety of stimuli. Nevertheless, few studies have considered dynamic indices, such as conditioned pain modulation (CPM), temporal summation of pain (TSP) or offset analgesia (OA), which are more closely linked to the underlying neurobiological mechanism of pain.^7^ In this line, it has been found that women who experienced chronic pain after breast cancer surgery, as compared with women without pain after that surgery, displayed enhanced TSP of mechanical pain (ie, increased painful sensations with repeated stimulation) and deficits in CPM.^8 9^ These studies suggest that persistent postoperative pain may be associated with alterations in endogenous pain inhibition at the central nervous system and that treatment strategies should target those pain-modulatory systems. Given that demographic factors such as age^10^ and sex^11 12^ influence endogenous pain modulation, the role of these variables as potential moderators of cancer pain biomarkers should be studied.

On the other hand, the assessment of EEG in resting state^13^ and in response to noxious stimuli^6^ may contribute to characterising increased/modified brain responsiveness to nociceptive stimulation in patients with cancer. The pain experience is the product of specific patterns of neural activity, but it is largely unknown how it is represented in the brain in oncological patients and how it can be modulated or mitigated. Neural correlates of pain at the scalp level are observed as modifications in oscillatory electrical activity, particularly within theta, alpha and gamma frequency bands, that are associated with activity in areas like the operculoinsular cortex or the primary somatosensory cortex.^14^ When a nociceptive stimulus is perceived as painful, power increases and decreases at those bands and cortical locations can be observed. In addition, these oscillatory components (specifically theta and gamma) appear to be intimately related, since the high-frequency gamma oscillations appear embedded in specific phases of theta, a mechanism thought to be involved in the integrated perception of pain.^15^

Moreover, EEG allows the extraction of indices of pain processing with excellent temporal resolution. In this case, the contact heat evoked potentials (CHEPs) are obtained during the presentation of noxious heat stimuli. CHEPs can be observed over central-parietal scalp areas contralateral to the stimulated hand (N1 wave) as well as a biphasic negative-positive complex maximal at the vertex and peaking from 190 to 400 ms (N2 and P2 components) after stimulus presentation.^16 17^ To our knowledge, no previous studies have analysed electrical brain activity during the presentation of nociceptive stimulation in patients with cancer.

### Objectives

The primary objective of this observational study is to assess a panel of central biomarkers of pain modulation (QST and EEG) and select the best classifiers to profile patients with pain in relation to those who do not experience pain. Secondary objectives are:

- To create a database of pain modulation biomarkers in cancer pain, providing a simple, standard protocol, guidelines and normative data.
- To analyse the sensitivity of the biomarkers used, and the role of other moderator variables such as age and sex.
  Our hypotheses are the following:
  - The study of diagnostic biomarkers derived from QST and EEG will help to characterise the patients with cancer pain according to the mechanisms of pain perception and/or modulation.
  - The central pain modulation mechanisms will be more altered in older patients and in females than in males.
  - Using machine learning algorithms, it will be possible to stratify patients with cancer pain based on the pain biomarkers.

## Methods

### Study settings

This is a multicentre study performed in six countries: Spain, Germany, Switzerland, Romania, Israel and Czech Republic. Nine research centres/clinical units (SERGAS-IDIS, SERGAS-IISGALICIASUR, USC, TECHNION, UMG, EOC, IOB, HCS, UHHK) will be involved. The centres have been selected due to their expertise in chronic pain, cancer, QST or EEG as well as their commitment to the project, and their capacity to recruit the target population while ensuring harmonised implementation of the study protocol across all sites. This study will be led by SERGAS through IDIS (Institute of Health Research in Santiago de Compostela, Spain), in coordination with USC.

### Study design and population

This is a cross-sectional, case–control, multicentric study to validate the panel of biomarkers involving a minimum of 470 ambulatory patients with cancer. The study aims to recruit at least 313 non-hospitalised patients with cancer pain (cases) and 157 non-hospitalised patients with cancer and no oncological pain (controls). Cancer pain is defined as pain directly attributable to the cancer disease or its treatment (eg, primary tumour, metastases, cancer surgery, systemic therapies or radiotherapy), as determined by the healthcare professionals. Five types of cancer (lung, breast, pancreas, prostate and colon) will be recruited. All the patients will be screened according to the inclusion and exclusion criteria presented in table 1. Participants with neurological or severe psychiatric disorders (except anxiety or depression) are excluded because these conditions may influence pain processing and neurophysiological measures, potentially confounding the interpretation of the study biomarkers.^18^

**Table 1:** Study eligibility criteria

Cases	
Inclusion criteria	Exclusion criteria
Adult subjects≥18 years old. • Able to provide informed consent to participate in the study. Able to self-report pain. To have a diagnosis of cancer affecting lung, breast, pancreas, prostate or colon (with metastases or not). Life expectancy of at least 6 months. Cancer pain persisting despite best tolerated pharmacological treatment. Oncological pain rated ≥5 on a 0–10 Numeric Rating Scale (NRS) persisting for over 1 month prior to enrolment.* Ability to use internet and WhatsApp or have a person (relative, caregiver) to help them.	Pregnant women or women of fertile age that did not have efficacious contraception during the whole period of the study. Neurological or psychiatric diseases (except anxiety or depression). Unstable medical conditions (eg, uncontrolled diabetes, uncompensated cardiac issues, heart failure or chronic obstructive pulmonary disease). History of neurosurgery, traumatic brain injury with loss of consciousness and/or cortical lesions.
Controls	
Inclusion criteria	Exclusion criteria
Adult subjects≥18 years old. Able to provide informed consent to participate in the study. Able to self-report pain. To have a diagnosis of cancer affecting lung, breast, pancreas, prostate or colon (with metastases or not). Life expectancy of at least 6 months. Ability to use internet and WhatsApp or have a person (relative, caregiver) to help them.	Pregnant women or women of fertile age that did not have efficacious contraception during the whole period of the study. Neurological or psychiatric diseases (except anxiety or depression). Unstable medical conditions (eg, uncontrolled diabetes, uncompensated cardiac issues, heart failure or chronic obstructive pulmonary disease). History of neurosurgery, traumatic brain injury with loss of consciousness, and/or cortical lesions. Chronic pain after cancer disease or treatment or oncological pain≥4 for more than 1 month.*

* Participants who otherwise meet all eligibility criteria but report an average NRS pain score of 4 during the previous month will be re-evaluated after 1 month and subsequently, if necessary, until they meet the eligibility criteria for either the case or the control group.

Participant recruitment and data collection commenced in May 2023 and are currently ongoing. Recruitment is expected to continue until November 2026.

Using G*Power (V.3.1.9.7), setting alpha at 0.05 and power (1-β) at 0.95, we estimated a final sample of 313 participants with pain (cases) and 157 participants with no pain (controls) to get a two-tailed small effect size of |Δ slope|=0.025 in a linear bivariate regression model.

The analysis will include eight predictors, selected based on the most sensitive variables identified in a complementary ongoing study.^19^ Therefore, the sample size was calculated to be adequate for logistic regression analysis with eight predictors in the chronic pain participants (following the formula n=100+50 i, where 50 refers to the event per variable, and *i* refers to dependent variables in the final model).^20^ Although these calculations indicate that a minimum sample of 470 participants would be sufficient to achieve adequate statistical power, additional participants are expected to be recruited according to the recruitment capacity of the participating units, providing some margin for potential loss of usable data due to technical issues or incomplete or invalid QST or EEG recordings.

### Enrolment of participants

A convenience sample of eligible ambulatory patients will be recruited from the oncology services or pain units of the hospitals. The physicians or therapists working in Pain Units, Oncology and Palliative care services will identify potentially eligible patients and present the study to them. Patients who meet the predefined inclusion and exclusion criteria (table 1) and agree to participate will provide written informed consent and will then be allocated to the case or control group according to the study eligibility criteria. In addition, alternative recruitment strategies will be employed, such as through oncology patient associations and social media. Once patients have signed the informed consent, the researchers of the study will contact them to carry out the assessment.

### Patient and public involvement

Patient and public involvement (PPI) was included in the design of this study. Representatives of patients with cancer-related pain contributed to the development of the study protocol, including feedback on study procedures. PPI will also be incorporated into the dissemination plan, ensuring that study findings are communicated in a clear and accessible manner to patient communities.

No patients or members of the public were involved in the conduct or analysis of this protocol beyond this stage.

### Assessment

The assessment of participants (cases and controls) will consist of a face-to-face session with an estimated duration of 2 hours. Participants will complete an exhaustive assessment protocol, which includes an interview (sociodemographic, clinical and health-related and lifestyle variables), QST and EEG recordings. Questionnaires will be completed online through the PAINLESS platform. In addition, clinical history data will be collected. The variables collected, tests and questionnaires completed in the evaluation are shown in table 2.

**Table 2:** Variables collected and questionnaires used

Sociodemographics (interview)	Age, gender, weight, height, education, occupation, marital status
Clinical variables (interview and clinical history)	Type of cancer, cancer treatment, comorbidities, surgeries, medication, family history, menstrual cycle, life habits, subjective and objective isolation, pain (type, frequency, chronology, expectations related to pain), condition 24 hours before assessment (hours of sleep, medication intake, etc.) and other relevant information (see online supplemental annex 1).
Questionnaires	- Brief Pain Inventory (BPI)
	- Numeric Rating Scale (NRS): Cancer Pain Intensity, Distress and Interference
	- 36-Item Short Form Survey (SF-36)
	- 3-level version of EQ-5D (EQ-5D-3L)
	- Modified Fatigue Impact Scale (MFIS)
	- Pain Catastrophizing Scale (PCS)
	- Patient Health Questionnaire-9 (PHQ-9)
	- General Anxiety Disorder-7 (GAD-7)
	- Sleep Scale from the Medical Outcomes Study (MOS-SS)
QST	- Temporal summation of pain (TSP)
	- Conditioned pain modulation (CPM)
	- Heat pain threshold (HPT)
	- Cold detection threshold (CDT)
	- Cold pain threshold (CPT) - Offset analgesia (OA) (optionally)
EEG	- Resting state electroencephalography (EEG)
	- Resting state EEG during cold pain
	- Contact heat evokedpotentials (CHEPS)

QST, quantitative sensory testing.

#### Questionnaires

Based on the IMMPACT-II (Initiative on Methods, Measurements and Pain Assessments in Clinical Trials-II) consensus meeting, participants will fulfil the following questionnaires to measure different variables: pain, fatigue, quality of life, quality of sleep, emotional functioning and catastrophism.^21^

- *Numeric Rating Scale (NRS) of cancer pain intensity, pain unpleasantness and interference due to pain (referred to the last week).* Three 11-point NRS will be used for pain intensity (from 0—‘no pain’ to 10—‘intolerable pain’), unpleasantness caused by pain (from 0—‘no unpleasant at all’ to 10—‘maximum unpleasantness of pain’) and interference of oncological pain in daily life (from 0—‘no interference at all’ to 10—‘maximum interference’). Measuring these affective and functional dimensions alongside pain intensity aligns with the core outcome domains recommended by the IMMPACT consensus for clinical pain protocols.^21^
- *Brief Pain Inventory (BPI**)*: the BPI consists of 12 items that measure the sensory and reactive dimensions of pain, severity or intensity of pain and its level of interference with several aspects of daily life. Interference is divided into activity and affective subdimensions. The BPI also includes a body diagram that allows participants to indicate the anatomical distribution of their pain. A recall period of 7 days or 24 hours is used.^22^
- *Pain Catastrophizing Scale (PCS**)*: the PCS is a 13-item self-report measure of catastrophising in the context of actual or anticipated pain. The PCS measures catastrophising as a multifaceted construct with three subscales: rumination, magnification and helplessness.^23^
- *36-item Short-Form Health Survey (SF-36)*: the SF-36 is used to assess patient health. It consists of items or questions that present respondents with choices about their perception of their health. The SF-36 includes one multi-item scale that assesses eight health concepts: (1) limitations in physical activities because of health problems; (2) limitations in social activities because of physical or emotional problems; (3) limitations in usual role activities because of physical health problems; (4) bodily pain; (5) general mental health (psychological distress and well-being); (6) limitations in usual role activities because of emotional problems; (7) vitality (energy and fatigue) and (8) general health perceptions. A recall period of a 4-week time frame is used. Higher scores are associated with a better quality of life.^24^
- *EQ-5D-3L*: The EQ-5D-3L was developed by the EuroQol Group. The EQ-5D-3L essentially consists of the EQ-5D descriptive system and the EQ visual analogue scale (EQ VAS). The EQ-5D descriptive system is composed of five items: ‘mobility’, ‘self-care’, ‘usual activities’, ‘pain/discomfort’ and ‘anxiety/depression’ assessed at three levels of description. The outcome describes the patient’s health state. The EQ VAS records the patients’ self-rated health on a vertical visual analogue scale where the endpoints are labelled ‘Best imaginable health state’ and ‘Worst imaginable health state’. The VAS can be used as a quantitative measure of health outcome that reflects the patients’ own judgement.^25^
- *Modified Fatigue Impact Scale (MFIS**)*: the MFIS was designed as a self-report measure to assess fatigue and how it affects the daily lives of patients with multiple sclerosis. This instrument provides an assessment of the effects of fatigue in terms of physical, cognitive and psychosocial functioning. The MFIS consists of 21 items: 9 ‘physical’ items, 10 ‘cognitive’ items and 2 ‘psychosocial’ items. Higher scores indicate a greater impact on quality of life. This questionnaire asks respondents to reflect on their experiences over the past 4 weeks.^26^
- *Patient Health Questionnaire (PHQ-9)*: the 9-item depression module from PHQ-9 is widely used as an open-access screening instrument for depression. The PHQ-9 asks patients to rate, on a 4-point scale ranging from ‘not at all’ to ‘most days’, the frequency with which they have experienced certain depression symptoms in the preceding 2 weeks.^27^
- *Generalised Anxiety Disorder Assessment (GAD-7)*: the GAD-7 is an open-access self-report anxiety questionnaire designed to assess the patient’s health status during the previous 2 weeks. This is a questionnaire consisting of 7 items, each of them being scored as 0 (not at all), 1 (several days), 2 (more than half the days) and 3 (nearly every day).^28^
- *Sleep Scale (SS) from the Medical Outcomes Study (MOS-SS)*: the MOS-SS provides subjective information on the quality and quantity of sleep over the previous 4-week period. It consists of a 12-item questionnaire that aims to evaluate key constructs of sleep, with derived subscales for the domains of sleep disturbance (4 items), quantity of sleep (1 item), snoring (1 item), awakening due to short of breath or headache (1 item), sleep adequacy (2 items) and somnolence (3 items).^29^
- *Eastern Cooperative Oncology Group (ECOG) Performance Status Scale*: the ECOG is widely used to evaluate the functional status of patients with cancer and their prognosis. The ECOG score ranges from 0 (fully active) to 5 (dead).^30^

#### QST and EEG—pain biomarkers

For QST assessment, heat and cold stimuli will be applied using the thermal contact stimulator (TCS II) developed by QST.LAB (https://www.qst-lab.eu/tcs-technical-description). The TCS II stimulator enables precise measurement of the threshold due to its ability to reach the target temperature in a few milliseconds. Indeed, this device is composed of several micro-Peltier thermoelectric elements, which enable very steep temperature ramps (ie, up to 300°C/s).

The dominant volar forearm will be used for: TSP, CPM, heat pain threshold (HPT), cold detection threshold (CDT), cold pain threshold (CPT), CHEPS—during EEG recording, and OA. The non-dominant volar forearm will be used for familiarisation and establishment of the Pain5 value (intensity of stimuli associated to a NRS of 5/10) to avoid sensitisation of the dominant forearm. For the resting-state EEG recording during cold pain, we will use the palm of the dominant hand. If the volar forearm and palm of the dominant hand are primary pain sites, the procedure should be applied to the non-dominant arm counterparts. If this is also considered a primary site of pain, the dorsum of the forearm or hand will be used instead.

### Procedure (in order of application)

The protocol followed is summarised below; for further technical details, the full procedure can be consulted in the study by Carrillo de la Peña *et al*.^19^ The perceived pain intensity to ongoing thermal stimulations will be assessed using a NRS from 0 to 10. First, participants will undergo familiarisation with the cold and heat stimuli. Then, we will calculate the Pain5, defined as the temperature resulting in pain intensity ratings of 5 out of 10 on the NRS scale. Two determination procedures will be repeated two times (each): (1) Pain5 for TSP and (2) Pain5 for CPM and OA. Both Pain5 measures would be calculated as the mean of the two repetitions for each determination procedure. If the pain intensity rating is higher than 5/10, we will reduce the temperature by −1 °C (ie, 44 °C) for subsequent stimulation. Conversely, if the pain intensity rating is lower than 5/10, the temperature will increase by +1 °C (ie, 46°C) for the next stimulation. We will repeat this process until a pain intensity rating of 5/10 is reached in a constant way.

### Temporal summation of pain

Initially, a single stimulus at the determined ‘Pain5 for TSP’ temperature is applied. The participant is asked to rate the pain intensity for this stimulus (time to rate 10s). Then, 10 stimuli at the determined ‘Pain5 for TSP’ temperature will be applied and the participant is asked to rate the pain intensity for the last of the stimuli in the sequence. The TSP index will be calculated as the normalised difference in pain intensity ratings between the initial single stimulus and the last pulse of the 10 stimulus sequence.

### Conditioned pain modulation

CPM requires the use of two different stimuli. (1) The test stimulus (TS) is the ‘Pain5 for CPM and OA’ temperature, applied with a 10s duration. (2) The conditioning stimulus (CS) is applied by having the participant rest the palm of their non-dominant hand on a cold plate at 10°C (CPM Plate, QST.LAB). First, the TS is applied, and participants are requested to rate the evoked pain intensity on an NRS. Then, participants are asked to place the palm of their non-dominant hand on the cold plate and when the hand has been on the cold plate for 20s, the participant is asked to rate the pain intensity. When the hand has been on the cold plate for 30s, the TS is reapplied, and the participant is asked to rate the pain intensity evoked by this TS stimulus using the NRS. As soon as the evoked pain intensity for this second TS application (ie, ‘conditioned TS’) has been produced, the palm of the non-dominant hand is removed from the cold plate.

CPM index is calculated as the normalised difference between the evoked pain intensity ratings for the ‘conditioned TS’ and the first application of the TS (ie, ‘unconditioned TS’).

### Heat pain threshold, cold detection threshold and cold pain threshold

Thresholds will be estimated using the method of limits as described by Rolke *et al*.^31^ For each threshold (HPT, CDT and CPT) we will perform three trials. For the estimation of HPT and CPT, the participant should press the response button as soon as the thermal sensation (heat for HPT and cold for CPT) starts to become painful. For the CDT estimation, the participant is asked to press the response button as soon as cold is perceived. The arithmetic means of the temperature at response button press across the three trials for each threshold will be used as the corresponding threshold estimation.

### Electroencephalogram

After the QST, EEG recording will be performed. This procedure allows the recording of brain activity. A portable, adaptable and simple to use recording system developed and adapted to clinical use by MENTALAB (Mentalab GmbH, Munich, Germany) will be employed. EEG signals will be recorded by 32 electrodes mounted in an elastic cap and arranged according to the international 10–20 system. Moreover, an ECG recording will be performed using one electrode. The EEG signal will be amplified and digitised using a sampling rate of 250 Hz and the reference will be placed at CPz. Impedances will be kept under 10 kΩ for all electrodes. The EEG session comprises recording of brain activity in three different situations: *(a) resting state EEG*: we will record 5 min with eyes closed. The participant will be instructed to sit comfortably and relax, letting their mind wander. (*b) resting state EEG during cold pain*: we will record 5 min with eyes closed. The participant will be instructed to sit comfortably and relax, letting their mind wander while a cold, painful stimulus is applied to the palm of their dominant hand. At the end of the test, the participant will be asked to rate how intense the painful stimulus was during the 5 min on average. The intensity of the cold painful stimulus has to be between 3 and 5/10 on the NRS during the whole recording. (*c) CHEPS*: will be recorded to obtain a neural correlate of nociceptive processing with high temporal resolution, using the TCS-II device. Stimulation location will be moved slightly between each stimulus application to avoid habituation of the thermal receptors. We will apply 20 stimuli between 55°C and 60°C with a duration of 0.3 s, with an interstimulus interval of 10s, to minimise saliency and startle responses. Quantitative EEG analytical techniques will enable evaluation of changes in spectral power and intersite phase connectivity.^14^

After the EEG recordings, the final QST procedure–OA–will optionally be performed, depending on whether each clinical unit decides to apply it.

### Offset analgesia

OA is defined by a disproportional decrease in perceived pain intensity after a slight decrease in a painful heat stimulus.^32^

First, a familiarisation step will be done with the stimulation intensity set at the determined ‘Pain5 for CPM and OA’ temperature minus 2°C. Participants will be asked to use the NRS to rate the perceived pain intensity evoked by this stimulation. OA will be induced by applying the determined Pain5 for CPM and OA temperature during 5s, then this temperature plus 1°C for another 5 s, and then again, the determined Pain5 for CPM and OA temperature for 20 s. A constant heat stimulus with the determined Pain5 for CPM and OA temperature for 30 s will be used as control stimulus. It will be applied either after or before the OA stimulus according to a counterbalancing of the sample scheme. The patients will rate the perceived pain intensity continuously on an Electronic Visual Analog Scale (QST.LAB) during both stimulation paradigms (OA and control). OA index is calculated as the normalised difference in pain intensity ratings during the last 20 s of the OA trial and the control trial.

### Outcomes

This study aims to stratify patients with cancer according to the presence or absence of pain, in order to identify the variables and characteristics that differ between these groups. This information may contribute to a better understanding and classification of pain mechanisms in this population, using QST as the main assessment tool.

### Data collection, management and analysis

#### Data collection and management

Patients’ data will be collected in the PAINLESS online platform. It facilitates collaborative work and is an optimal tool for clinical research. The Data Collections will be pseudonymised, protecting the participant’s identity. The research team and the health authorities, who have a duty of confidentiality, will have access to all data collected only in their clinical site.

The Data Management Plan includes the following aspects: data outputs, findability of data, accessibility, Intellectual Property Rights (IPR) considerations, provisions for access to restricted data for verification purposes, interoperability (standards, formats and vocabularies for data and metadata), reusability and storage.

The data will be stored using the infrastructure of the Supercomputing Centre of Galicia (CESGA) with regular backups. There will be a training session on data management for the consortium members. Regarding data analysis, we have partnered with experts in statistics and machine learning analyses.

#### Data analysis

The statistical analysis will be structured according to the three study hypotheses, using complementary statistical and machine learning approaches to assess biomarker-based classification, the influence of demographic factors on central pain modulation, and the characterisation of patients with cancer pain.

- Hypothesis A:
  Patients will be classified according to clinically significant pain status using a prespecified panel of QST and EEG biomarkers. A multivariable logistic regression model with Firth’s penalty and intercept correction will be used as the primary classification model. Validation will be performed using repeated stratified cross-validation, with data imputation and scaling performed within the training folds. Model performance will be assessed using the area under the receiver operating characteristic curve, calibration and precision–recall curves. As a secondary analysis, individual biomarkers will be evaluated using separate Firth logistic regression models adjusted for age and sex, with Benjamini–Yekutieli correction for multiple comparisons.
- Hypothesis B:
  The association between age, sex and central pain modulation measures will be assessed using linear regression when a single measurement per patient is available. Age, sex and their interaction will be included as explanatory variables. In a complementary analysis, clinically significant pain status will be modelled as the outcome using Firth logistic regression, with pain modulation measures, age, sex and their corresponding interactions included as predictors.
- Hypothesis C:
  Machine learning approaches will be used to characterise patients with cancer pain using the multimodal biomarker database. A supervised pipeline will use elastic-net penalised logistic regression as the primary algorithm, with random forest and gradient-boosted trees as comparator methods. Model performance will be assessed using nested cross-validation and area under the receiver operating characteristic curve, calibration and precision–recall curves. An unsupervised pipeline will be used to identify patient profiles using a Gaussian mixture model fitted to prespecified continuous features. The number of profiles will be selected according to the Bayesian Information Criterion, and profile stability will be assessed using the adjusted Rand index.

## Ethics and dissemination

This study received ethical approval from the following local ethics committees: SERGAS-IDIS, SERGAS-IISGALICIASUR and USC: Territorial Research Ethics Committee of Santiago-Lugo (approval reference CEI 2022/324); TECHNION: Institutional Helsinki Committee of Rambam Health Care Campus (approval reference RMB0141-23); UMG: Ethics Committee of the Universitätsmedizin Göttingen (approval reference 17/1/24; DMIDS application number 00015102) with notification to the Competent Authority—Bundesinstitut für Arzneimittel und Medizinprodukte; EOC: Cantonal Ethics Committee of Ticino (approval reference CE 4287; BASEC 2023-D0009) and competent authority Swissmedic; HCS: Research Ethics Committee of the Hospice Casa Speranței Foundation (approval reference 03/24.08.2023); IOB: Scientific Research Ethics Committee of the Institute of Oncology ‘Prof. Dr. Al. Trestioreanu’ Bucharest (approval reference 1119/19.01.2023); and University Hospital Hradec Králové: Ethics Committee of the University Hospital Hradec Králové (approval reference 2 02 602 P06).

Moreover, it was registered prospectively in ISRCTN (International Standard Randomized Controlled Trial Number) registry, Trial ID: ISRCTN35887998 (https://www.isrctn.com/ISRCTN35887998). It is a primary clinical trial registry recognised by WHO and ICMJE that accepts all clinical research studies. This registry provides content validation and the unique identification number necessary for publication. All study records in the database are freely accessible and searchable.

In addition, this study does not involve any invasive or dangerous procedures. Regarding the safety of study participants, a trained investigator will administer the sensory test and other evaluations. Prior to performing any study procedures, the study staff member will be trained in the techniques of QST, EEG and questionnaire administration.

Results will be disseminated through publication in peer-reviewed journals in the fields of cancer and pain and through presentations at national and international conferences. The anonymity of all participants will be strictly maintained, and all data will be handled in compliance with relevant data protection laws, including the GDPR.

To guarantee secure data curation and preservation, and whenever possible, accessibility to project data by other potential users. We will follow the FAIR data principles (Findability, Accessibility, Interoperability, and Reusability) and will use already existing infrastructures (eg, the EUDAT for data management, European Open Science Cloud (EOSC), European Data Infrastructure (EDI)).

## Supplementary material

10.1136/bmjopen-2026-121471 online supplemental file 1
