# Supplementary material for: Determination of central pain modulation biomarkers to characterise patients with cancer and refractory pain: protocol for a cross-sectional study
Source: BMJ Open. 2026 Oct 7;16(10):e121471. doi: 10.1136/bmjopen-2026-121471 (PMC13646981; doi:10.1136/bmjopen-2026-121471)
Supplement: Supplementary file 1 [file bmjopen-2026-121471.reviewer_comments.pdf]

PEER REVIEW HISTORY

BMJ Open publishes all reviews undertaken for accepted manuscripts. Reviewers are asked to complete a checklist review form and are provided with free text boxes to elaborate on their assessment. These free text comments are reproduced below.

ARTICLE DETAILS

Title (Provisional)

Determination of central pain modulation biomarkers to characterize patients with cancer and refractory pain: protocol for a cross-sectional study.

Authors

Rubal-Otero, Lara; Martinez-Rodriguez, Natalia; Álvarez-Rodríguez, Margarita; González-Villar, Alberto; Consortium, PAINLESS; Carrillo-de-la-Peña, María T

VERSION 1 - REVIEW

**Reviewer**	**1**
**Name**	**Patil, Deepali**
**Affiliation**	**Ravi Nair Physiotherapy College**
**Date**	**22-May-2026**
**COI**	**None**

References should be more than 25. Three hypotheses are given in this study so accordingly the statistical analysis will change. The author taken NRS for pain. they can take NPRS for pain of patient. CONSORT flow chart should be there for study method understanding.

**Reviewer**	**2**
**Name**	**Powell, Victoria**
**Affiliation**	**University of Michigan, Division of Geriatric and Palliative Medicine**
**Date**	**22-Jul-2026**
**COI**	**None**

Title: Needs to specify “Protocol” in title

Abstract: Need to more clearly indicate this is a planned study / protocol and no data has been collected.

Introduction: The authors rely on citations that are very old and for which more updated literature is available. For example, cancer pain prevalence estimates have been updated since the 2003 Bruera study.

Terminology for pain mechanisms is also outdated. Nociceptive pain would encompass what authors refer to as “inflammatory” pain; Nociplastic is the preferred terminology for what was previously called “central pain” or “central sensitization.” (Fitzcharles M, Cohen S, Clauw D et al. Nociplastic pain: towards an understanding of prevalent pain conditions. The Lancet, 397, 2098-2110).

Hypothesis b: What is the authors’ justification that central pain modulation mechanisms will be more dysfunctional in older patients and females? Moreover, what is the definition of “dysfunctional” central pain modulation mechanisms?

Hypothesis c: Do authors mean data for machine learning algorithm training?

Methods:

Justification for why the study is performed at the specific locations is inadequate; there are no citations or rationale given. Are the patient populations particularly representative somehow, or is there some other reason? Please explain.

There is no justification for how the cases and controls will be allocated. Strongly suggest that authors review a tool to reduce bias in sample selection such as the Newcastle-Ottawa Scale. https://ohri.ca/en/who-we-are/core-facilities-and-platforms/ottawa-methods-centre/newcastle-ottawa-scale

Page 7, line 40-41. Why would dropouts affect a cross-sectional study? How could someone dropout of a cross-sectional study? It would seem either they provide data, or they do not. Please explain.

“Cancer pain” is not clearly defined. Please define what is meant by cancer pain - is this pain from oncologic surgeries (e.g. post-operative pain), pain related to other treatment (e.g. radiation-induced mucositis), or pain related to tumor invasion/growth/metastases, or all of the above?

Why are patients with neurologic or psychiatric disease excluded? There is no rationale and ethically this is not justifiable as it is currently written. Some rationale needs to be given.

Table 1: One of the primary differentiating factors between cases and controls is the NRS, which is a single-item scale. Participants rating pain 0-3 would be allocated to “controls” while those rating pain 5-10 would be allocated to "cases." What would happen should a participant rate pain a those rating pain a "4" ?

Section 2.3 Enrollment: Needs to more clearly specify “convenience sample” in the design.

Section 2.5.1 The NRS is not commonly used to refer to any variables other than pain intensity. Justification for using a Likert type 0-10 rating scale for factors other than pain intensity, such as interference, should be provided along with citations.

Widespread pain, accompanying central nervous system symptoms (depression, unrefreshing sleep, fatigue) are key features of nociplastic pain/central pain. There is no measure of pain widespreadedness. Authors will be limited in the conclusions they are able to draw about the presence of central pain if a pain widespreadedness measurement, such as the Widespread Pain Index, a Body Map, or other measurement) (Brummett et al, Preliminary validation of the Michigan Body Map. Pain. 2016 Jun;157(6):1205-1212.)

There are no dates provided for when data collection will occur. Has it started already? When is completion planned? How long will recruitment take?

VERSION 1 - AUTHOR RESPONSE

Reviewer 1

Comment 1

Comment: *References should be more than 25.*

Response: We thank the Reviewer for this suggestion. We have revised and updated the reference list by incorporating several recent and relevant publications throughout the Introduction, particularly in relation to cancer pain epidemiology, current pain taxonomy, quantitative sensory testing, pain modulation mechanisms, electroencephalography biomarkers, and factors influencing pain modulation. These additions strengthen the scientific background of the study and increase the overall number of references included in the manuscript, from 25 to 32.

Changes made in the manuscript:

- 1. Introduction – Section 1.1. Background (Pages 4–5).
- 2. Methods – Section 2.2. Study design and population (Page 6).
- 4. Full references (Pages 17–19).

Comment 2

Comment: *Three hypotheses are given in this study so accordingly the statistical analysis will change.*

Response: We agree that the statistical analysis should be clearly aligned with the three hypotheses. The Statistical Analysis section has therefore been revised to specify the analytical approach for each hypothesis. Hypothesis A will be assessed using multivariable logistic

regression with Firth's penalty and intercept correction, with repeated stratified cross-validation and appropriate performance metrics. Hypothesis B will be assessed using linear regression to examine the association between age, sex, and central pain modulation measures, including their interaction, together with complementary logistic regression with Firth's penalty to examine the relationship between pain modulation measures, age, sex, and clinically significant pain status. Hypothesis C will be addressed using supervised and unsupervised Machine Learning approaches to assess whether patients with cancer pain can be stratified based on their pain biomarkers. The supervised pipeline will use elastic-net penalised logistic regression as the primary algorithm, with random forest and gradient-boosted trees as comparator methods, while the unsupervised pipeline will use Gaussian mixture modelling to identify patient profiles. The corresponding performance and stability metrics have also been specified.

Changes made in the manuscript: 2. Methods – Section 2.7.2 Data analysis (Page 15-16).

Comment 3

Comment: *The author taken NRS for pain. They can take NPRS for pain of patient.*

Response: The Numeric Rating Scale (NRS) used in this study corresponds to the 11-point numerical pain rating scale commonly referred to in the literature as the Numeric Pain Rating Scale (NPRS). Both terms refer to the same validated instrument for assessing pain intensity. We agree that the term *NPRS* is more specific, as it explicitly refers to pain assessment. However, *NRS* is also widely used in the pain literature, including in the IMMPACT recommendations, and has been used consistently throughout the study protocol. Thus, we have decided to retain this terminology to ensure consistency across the manuscript and study documentation.

Changes made in the manuscript: No changes were made to the manuscript.

Comment 4

Comment: *CONSORT flow chart should be there for study method understanding.*

Response: As this study is an observational case-control protocol rather than a randomized controlled trial, a CONSORT flow diagram is not applicable. The participant recruitment and enrolment process is described in detail in Section 2.3 (Enrolment of participants), including eligibility screening, informed consent and allocation to the case and control groups according to the predefined eligibility criteria. Therefore, we believe that the current description adequately explains the study methodology. In the future published paper, we will follow the STROBE statement for observational case-control studies. This has been included in the Abstract page.

Changes made in the manuscript: Abstract- Ethics and dissemination (Page 2).

Reviewer 2

Comment 1

Comment: *Title: Needs to specify "Protocol" in title.*

Response: We thank the Reviewer for this valuable suggestion. We agree that explicitly identifying the manuscript as a study protocol in the title improves clarity and accurately reflects the nature of the study. Accordingly, the title has been revised to include the term "protocol".

Changes made in the manuscript: Title (Page 1).

Comment 2

Comment: *Abstract: Need to more clearly indicate this is a planned study / protocol and no data has been collected.*

Response: We agree that the abstract should more clearly indicate that this manuscript presents a study protocol. The abstract has been revised to explicitly state that it describes the protocol of a planned multicentre case–control study.

Changes made in the manuscript: Abstract - Introduction and Methods and analysis (Page 2).

Comment 3

Comment: *Introduction: The authors rely on citations that are very old and for which more updated literature is available.*

Response: We have revised the Introduction to incorporate more recent and relevant literature supporting the rationale of the study. Specifically, we updated the epidemiological evidence on cancer pain prevalence using a recent systematic review and meta-analysis (Snijders et al., 2023), incorporated the current pain taxonomy (Fitzcharles et al., 2021), included recent evidence supporting the use of EEG as biomarkers of pain processing (Zis et al., 2022; Zebhauser et al., 2023), added a reference supporting the use of Offset Analgesia (OA) as a dynamic measure of pain modulation (Hermans et al., 2016), updated the evidence on Conditioned Pain Modulation (CPM) and Temporal Summation of Pain (TSP) in persistent postoperative breast cancer pain (Edwards et al., 2013; Tan et al., 2023), and incorporated additional evidence supporting the influence of age and sex on endogenous pain modulation (Hackett et al., 2020; Bartley & Fillingim, 2013; Umeda & Okifuji, 2023).

Changes made in the manuscript:

1. Introduction – Section 1.1. Background (Pages 4–5).

- Updated the epidemiological data on cancer pain prevalence by replacing the prevalence estimates from Bruera & Kim (2003) with more recent estimates from the systematic review and meta-analysis by Snijders et al. (2023).
- Updated the description of cancer-related pain mechanisms and incorporated the current concept of nociplastic pain (Fitzcharles et al., 2021).
- Revised the description of Quantitative Sensory Testing (QST) and updated the supporting reference (Martland et al., 2020).
- Added recent references supporting the use of electroencephalography (EEG) as biomarkers of pain processing and chronic pain (Zis et al., 2022; Zebhauser et al., 2023).
- Added a reference supporting the use of Offset Analgesia (OA) as a dynamic measure of pain modulation (Hermans et al., 2016).
- Updated the evidence supporting alterations in Conditioned Pain Modulation (CPM) and Temporal Summation of Pain (TSP) in persistent postoperative breast cancer pain and their association with altered endogenous pain modulation (Edwards et al., 2013; Tan et al., 2023).
- Added recent evidence supporting the influence of age and sex on endogenous pain modulation (Hackett et al., 2020; Bartley & Fillingim, 2013; Umeda & Okifuji, 2023).

Comment 4

Comment: *Terminology for pain mechanisms is also outdated. Nociceptive pain would encompass what authors refer to as "inflammatory" pain; Nociplastic is the preferred terminology for what was previously called "central pain" or "central sensitization".*

Response: We agree that the terminology describing pain mechanisms should be updated to reflect current pain taxonomy. Accordingly, we have deleted the term *inflammatory pain* and incorporated the concepts of *nociceptive, neuropathic and nociplastic pain mechanisms* according to current pain taxonomy, and included the recommended reference (Fitzcharles et al., 2021). We have also revised the description of central pain processing and pain modulation to better align with current terminology while preserving the focus of the study on pain processing and pain modulation.

Changes made in the manuscript: 1. Introduction - Section 1.1 Background (Page 4).

- Deleted "inflammatory pain" (Paragraph 1).
- Revised "oncological pain mechanisms are poorly understood" to "the mechanisms underlying cancer-related pain remain poorly understood" (Paragraph 1).
- Revised the description of pain mechanisms according to current pain taxonomy, introducing *nociceptive, neuropathic and nociplastic pain mechanisms* and adding the reference Fitzcharles et al. (2021) (Paragraph 1).
- Revised the opening sentence of the second paragraph from "It is possible to measure central pain processing and modulation mechanisms..." to "It is possible to assess central mechanisms involved in pain processing and pain modulation..." to better reflect current terminology (Paragraph 2).

Comment 5

Comment: *Hypothesis b: What is the authors' justification that central pain modulation mechanisms will be more dysfunctional in older patients and females? Moreover, what is the definition of "dysfunctional" central pain modulation mechanisms?*

Response: By the term "dysfunctional" we intended to refer to alterations in endogenous pain modulation, such as reduced inhibitory capacity (e.g., conditioned pain modulation) and/or enhanced facilitatory processes (e.g., temporal summation), rather than implying a specific pathological definition. We agree, however, that this wording may be ambiguous and could lead to different interpretations. Therefore, we have revised the hypothesis by replacing the term "dysfunctional" with "altered", which more accurately reflects the biomarkers evaluated in this study. We have also strengthened the Introduction by adding supporting evidence regarding the influence of age and sex on central pain modulation.

Changes made in the manuscript:

- 1. Introduction – Section 1.1. Background, third paragraph (Page 4): Justification for the influence of age and sex on central pain modulation.
- 1. Introduction – Section 1.2. Objectives (Page 5): Second hypothesis (replacement of *"dysfunctional"* with *"altered"*).

Comment 6

Comment: *Hypothesis c: Do authors mean data for machine learning algorithm training?*

Response: We thank the Reviewer for this helpful observation. Our intention was indeed to indicate that the biomarker database generated in this study could serve as a valuable dataset for the development of Machine Learning algorithms to stratify patients. Thus, the main point of the hypothesis is the possibility of proposing profiles of patients based on the pain biomarkers. As it was not clearly, we have reworded the hypothesis to clarify this point.

Changes made in the manuscript: Introduction – Section 1.2. Objectives, third hypothesis (Page 5).

Comment 7

Comment: *Justification for why the study is performed at the specific locations is inadequate; there are no citations or rationale given. Are the patient populations particularly representative somehow, or is there some other reason? Please explain.*

Response: The participating centres were selected considering the expertise of the research teams, their capacity to recruit the target population and their commitment to the project. We aimed to include teams from EU countries at various geographical points (N, S, E, and W Europe), to enhance the diversity of participating settings and the applicability of the data obtained across countries with different healthcare realities. As highlighted in the Introduction, cancer pain remains a widespread clinical challenge across healthcare settings, and the aim of

this multicentre study is to improve the understanding of cancer pain mechanisms in a broad population of patients with cancer using a harmonised study protocol. Note that we use a convenience sample, that does not intend to be representative. We have clarified this rationale in the manuscript.

Additionally, we would like to clarify that, during the revision period, a new partner, University Hospital Hradec Králové (Czech Republic), was officially incorporated into the study. At the time of the initial submission, its participation had not yet been formally confirmed. Its inclusion was subsequently considered necessary to further support participant recruitment, and the manuscript has therefore been updated accordingly.

Changes made in the manuscript:

1. Abbreviations (Page 3) and Methods – Section 2.1. Study settings (Page 6). New participating centre and updated number of centres/countries added.

2. Methods – Section 2.1. Study settings (Page 6). Rationale for centre selection clarified, including that the participating centres constitute a convenience sample.

Comment 8

Comment: *There is no justification for how the cases and controls will be allocated. Strongly suggest that authors review a tool to reduce bias in sample selection such as the Newcastle-Ottawa Scale.*

Response: We appreciate the Reviewer's suggestion regarding the Newcastle-Ottawa Scale and have clarified the participant recruitment and allocation procedure accordingly. First of all, it should be clarified that we use a "convenience sample", understood as a non-probability sampling method where researchers select readily available, accessible participants rather than choosing them randomly. The recruitment procedure was already described in the manuscript; however, we agree that the allocation of participants to the case and control groups could be explained more clearly. We have therefore revised the description of the enrolment process to explicitly state that physicians or therapists working in the participating Pain Units, Oncology and Palliative Care Services will identify potentially eligible patients, who will be then screened according to the predefined inclusion and exclusion criteria. Participants who meet these criteria and provide written informed consent will be subsequently allocated to the case or control group based on the study eligibility criteria. As a result, cases and controls are recruited from the same source population across the participating centres, thereby reducing the risk of selection bias.

Changes made in the manuscript: 2. Methods – Section 2.3. Enrolment of participants (Page 8).

Comment 9

Comment: *Page 7, line 40-41. Why would dropouts affect a cross-sectional study? How could someone dropout of a cross-sectional study? It would seem either they provide data, or they do not. Please explain.*

Response: We agree with the Reviewer that the inclusion of a 30% dropout rate was not appropriate for the present cross-sectional study, as there is no longitudinal follow-up. The term "dropout" was not used properly, as we meant "data loss", considering the possibility of problems in the assessments due to human errors, technical issues, incomplete or invalid QST or EEG recordings, fatigue of participants, or other problems occurring during data collection. We have therefore removed the dropout assumption and clarified that a minimum of 470 participants is required to achieve adequate statistical power. As we do not know in advance what percentage of data loss may occur, we decided to retain the sample size estimated with G*Power (313 cases and 157 controls). Nevertheless, additional participants may be recruited according to the recruitment capacity of the participating units to compensate for potential loss of usable data.

Changes made in the manuscript: Abstract - Methods and analysis (Page 2) and 2. Methods-Section 2.2. Study design and population (Page 6-7, paragraphs 1 and 4).

Comment 10

Comment: *"Cancer pain" is not clearly defined. Please define what is meant by cancer pain - is this pain from oncologic surgeries (e.g. post-operative pain), pain related to other treatment (e.g. radiation-induced mucositis), or pain related to tumor*

invasion/growth/metastases, or all of the above?

Response: We agree that the definition of cancer pain should be specified more clearly. In this study, cancer pain is defined as pain directly attributable to the cancer disease or its treatment (e.g., primary tumour, metastases, cancer surgery, systemic therapies or radiotherapy), as determined by the treating physician. The healthcare professional distinguishes cancer-related pain from pain due to other non-cancer-related conditions based on the clinical assessment of the patient. This clarification has been added to the manuscript.

Changes made in the manuscript: 2. Methods - Section 2.2 Study design and population (Page 6).

Comment 11

Comment: *Why are patients with neurologic or psychiatric disease excluded? There is no rationale and ethically this is not justifiable as it is currently written. Some rationale needs to be given.*

Response: We agree that the rationale for these exclusion criteria should be stated more explicitly. According to the available literature, neurological disorders and severe psychiatric disorders may independently alter pain processing, quantitative sensory testing (QST) responses and EEG activity, irrespective of cancer-related pain (Vaughan et al., 2019).

Consequently, these conditions could act as potential confounding factors for the biomarkers under investigation and compromise the interpretation of the study results. For this reason, they were included as exclusion criteria. Anxiety and depression were not excluded because they are highly prevalent among patients with cancer and constitute clinically relevant variables assessed within the study. This rationale has been incorporated into the manuscript.

Changes made in the manuscript: 2. Methods - Section 2.2 Study design and population (Page 6).

Comment 12

Comment: *Table 1: One of the primary differentiating factors between cases and controls is the NRS, which is a single-item scale. Participants rating pain 0-3 would be allocated to "controls" while those rating pain 5-10 would be allocated to "cases." What would happen should a participant rate pain a those rating pain a "4" ?*

Response: Participants with an NRS score of 4 at the time of screening will be not immediately allocated to either study group. Instead, they will be re-evaluated after one month until they meet the predefined eligibility criteria for either the case group (average pain intensity ≥5 during the previous month) or the control group (average pain intensity ≤3 during the previous month). We have clarified this point in the eligibility criteria to avoid ambiguity regarding participant allocation.

Changes made in the manuscript: 2. Methods – Table 1. Study eligibility criteria (Page 7-8).

Comment 13

Comment: *Section 2.3 Enrollment: Needs to more clearly specify "convenience sample" in the design.*

Response: We agree that the sampling strategy should be explicitly stated. Although the recruitment procedure was already described, we have now clarified that a convenience sample of eligible patients will be recruited from the participating centres.

Changes made in the manuscript: 2. Methods – Section 2.3. Enrolment of participants (Page 8).

Comment 14

Comment: *Section 2.5.1 The NRS is not commonly used to refer to any variables other than pain intensity. Justification for using a Likert type 0-10 rating scale for factors other than pain intensity, such as interference, should be provided along with citations.*

Response: We have clarified the rationale for using Numeric Rating Scales (NRS) to assess pain unpleasantness and pain interference, in addition to pain intensity. NRS were selected because they provide a rapid, simple and reliable method for obtaining direct quantitative ratings of different subjective dimensions of the pain experience through an 11-point numeric (Likert-type) scale. The selection of these pain domains is consistent with the recommendations of the IMMPACT-II consensus, which identifies pain intensity and pain-related functioning among the core outcome domains to be assessed in clinical pain research (Dworkin et al., 2005). The Brief Pain Inventory (BPI) is additionally included to provide a more comprehensive multidimensional assessment of pain interference in daily functioning, complementing the brief NRS assessment.

Changes made in the manuscript: 2. Methods - Section 2.5.1. Questionnaires. Numeric Rating Scale (NRS) subsection (Page 10).

Comment 15

Comment: *Widespread pain, accompanying central nervous system symptoms (depression, unrefreshing sleep, fatigue) are key features of nociplastic pain/central pain. There is no measure of pain widespreadedness. Authors will be limited in the conclusions they are able to draw about the presence of central pain if a pain widespreadedness measurement, such as the Widespread Pain Index, a Body Map, or other measurement).*

Response: We agree that the assessment of pain distribution is relevant when interpreting mechanisms related to central pain processing. Although this was not explicitly described in the manuscript, the Brief Pain Inventory (BPI) administered in this study includes a body diagram that allows participants to indicate the anatomical distribution of their pain. We have clarified this in the manuscript.

Changes made in the manuscript: 2. Methods – Section 2.5.1. Questionnaires. Brief Pain Inventory (BPI) subsection (Page 10).

Comment 16

Comment: *There are no dates provided for when data collection will occur. Has it started already? When is completion planned? How long will recruitment take?*

Response: We agree that providing a clear study timeline improves the transparency of the protocol. The manuscript has been revised to specify that participant recruitment and data collection commenced in May 2023 and are currently ongoing. We have also included the anticipated completion of recruitment, which is expected in November 2026.

Changes made in the manuscript: 2. Methods – Section 2.2 Study design and population (Page 6, paragraph 2). Study timeline updated.
