# Supplementary material for: Determination of central pain modulation biomarkers to characterise patients with cancer and refractory pain: protocol for a cross-sectional study
Source: BMJ Open. 2026 Oct 7;16(10):e121471. doi: 10.1136/bmjopen-2026-121471 (PMC13646981; doi:10.1136/bmjopen-2026-121471)
Supplement: Supplementary file 2 [file bmjopen-16-10-s001.docx]

**Supplementary Annex 1.** Clinical characteristics and sociodemographic variables.

**Clinical interview:**

- Sociodemographic data.
- Clinical history.
- Pain.
- Expectations related to pain.
- Family history.
- Current treatment.
- Menstrual cycle.
- LifeHabits (Tobacco, Alcohol, Coffee/ Tea, Other drugs, History of	addictions and Physical activity)
- Subjective and Objective Isolation

**Clinical history:**

- About	cancer.
  - Type	of tumor.
  - Date of diagnosis.
  - Metastasis.
  - Stage.
  - Beginning of the stage.
  - Surgery.
- About	cancer treatment
  - Oncological treatment
    - Start date of cancer treatment
    - End date of cancer treatment
    - Intention of treatment
    - Type of treatment
    - If neoadjuvant or palliative, response to treatment
    - Current line of treatment
      - Pancreatic cancer treatment
        - Chemotherapy
        - Radiotherapy
        - Others
      - Colon	cancer treatment
        - Chemotherapy
        - Target therapy
        - Radiotherapy
        - Others
      - Lung cancer treatment
        - Chemotherapy
        - Inmunotherapy
        - Target therapy
        - Radiotherapy
        - Others
      - Breast cancer treatment
        - Chemotherapy
        - Hormonotherapy
        - Inmunotherapy
        - Target therapy
        - Radiotherapy
        - Others
      - Prostate cancer treatment
        - Hormonotherapy
        - Chemotherapy
        - Radiotherapy
        - Inmunotherapy
        - Target therapy
        - Others
- About	oncological pain
  - Pain
  - Type of pain
  - Pain localization
  - The pain is related to
  - Non-opioid
  - Adjuvant
  - Second stage
  - Third stage
  - Interventional therapy
- Other	relevant information
